# Apatite-type SrPr_4_(SiO_4_)_3_O

**DOI:** 10.1107/S1600536810033349

**Published:** 2010-08-25

**Authors:** Terutoshi Sakakura, Minami Kamoshita, Hironaga Iguchi, Jun Wang, Nobuo Ishizawa

**Affiliations:** a Ceramics Research Laboratory, Nagoya Institute of Technology, Asahigaoka, Tajimi 507-0071, Japan

## Abstract

Single crystals of the title compound, strontium tetra­praseo­dymium tris­(silicate) oxide, SrPr_4_(SiO_4_)_3_O, have been grown by the self-flux method using SrCl_2_. The structure is isotypic with the apatite supergroup family having the generic formula ^IX^ *M*1_2_^VII^ *M*2_3_(^IV^ *T*O_4_)_3_ *X*, where *M* = alkaline earth and rare earth metals, *T* = Si and *X* = O. The *M*1 site (3.. symmetry) is occupied by Pr and Sr atoms with almost even proportions and is surrounded by nine O atoms forming a tricapped trigonal prism. The *M*2 site (*m*.. symmetry) is almost exclusively occupied by Pr and surrounded by seven O atoms, forming a distorted penta­gonal bipyramid. The Si atom (*m*.. symmetry) is surrounded by two O (*m*.. symmetry) and two O atoms in general positions, forming an isolated SiO_4_ tetra­hedron. Another O atom at the inversion centre (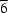.. symmetry) is surrounded by three *M*2 sites, forming an equilateral triangle perpendicular to the *c* axis.

## Related literature

Náray-Szabó (1930 ▶) and Mehmel (1930 ▶) independently determined the structure of fluorapatite for the first time. Since then, a large number of apatite supergroup minerals have been reported, as classified recently by Pasero *et al.* (2010 ▶). The synthetic rare-earth-bearing orthosilicates belonging to the apatite supergroup have attracted considerable attention since high oxide ionic conductivity was found (Nakayama *et al.*, 1995 ▶). The title compound is isostructural with CaLa_4_(SiO_4_)_3_O (Schroeder & Mathew, 1978 ▶).

## Experimental

### 

#### Crystal data

SrPr_4_(SiO_4_)_3_O
*M* *_r_* = 943.53
Hexagonal, 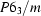

*a* = 9.5999 (1) Å
*c* = 7.1388 (1) Å
*V* = 569.76 (1) Å^3^
*Z* = 2
Mo *K*α radiation
μ = 21.82 mm^−1^
*T* = 296 K
0.14 × 0.05 × 0.03 mm

#### Data collection

Bruker APEXII CCD diffractometer
Absorption correction: multi-scan (*SADABS*; Sheldrick, 1996 ▶) *T*_min_ = 0.240, *T*_max_ = 0.546
31664 measured reflections
3151 independent reflections
2815 reflections with *I* > 2σ(*I*)
*R*_int_ = 0.030

#### Refinement

*R*[*F* ^2^ > 2σ(*F* ^2^)] = 0.018
*wR*(*F* ^2^) = 0.047
*S* = 1.07
3151 reflections
42 parameters
1 restraint
Δρ_max_ = 2.45 e Å^−3^
Δρ_min_ = −1.75 e Å^−3^

### 

Data collection: *APEX2* (Bruker, 2008 ▶); cell refinement: *SAINT* (Bruker, 2008 ▶); data reduction: *SAINT*; program(s) used to solve structure: *SHELXS97* (Sheldrick, 2008 ▶); program(s) used to refine structure: *SHELXL97* (Sheldrick, 2008 ▶) and *WinGX* (Farrugia, 1999 ▶); molecular graphics: *ATOMS for Windows* (Dowty, 2006 ▶); software used to prepare material for publication: *PLATON* (Spek, 2009 ▶) and *publCIF* (Westrip, 2010 ▶).

## Supplementary Material

Crystal structure: contains datablocks I, global. DOI: 10.1107/S1600536810033349/wm2391sup1.cif

Structure factors: contains datablocks I. DOI: 10.1107/S1600536810033349/wm2391Isup2.hkl

Additional supplementary materials:  crystallographic information; 3D view; checkCIF report

## Figures and Tables

**Table 1:** Selected bond lengths (Å)

Pr1—O1	2.4617 (8)
Pr1—O2^i^	2.5258 (9)
Pr1—O3^i^	2.8509 (12)
Pr2—O4	2.2809 (1)
Pr2—O3^i^	2.4296 (8)
Pr2—O2^ii^	2.4568 (12)
Pr2—O3^iii^	2.5519 (10)
Pr2—O1	2.6876 (15)
Si1—O1	1.6172 (12)
Si1—O2	1.6251 (13)
Si1—O3	1.6324 (9)

Symmetry codes: (i) 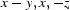; (ii) 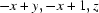; (iii) 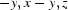.

**Fig. 1.**
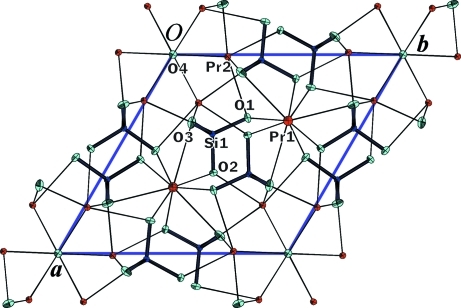
The crystal structure of SrPr4(SiO4)3O in projection along [001]. Pr and Sr atoms are given as red, Si as dark blue, and O atoms as light blue ellipsoids at the 50% probability level.

## supplementary crystallographic information

### Comment

Náray-Szabó (1930) and Mehmel (1930) independently determined the structure of fluorapatite for the first time. Since then, a large number of apatite supergroup minerals and synthetic compounds have been reported, as classified recently by Pasero *et al.* (2010). The apatite supergroup has a general formula ^IX^*M*1_2_^VII^*M*2_3_(^IV^*T*O_4_)_3_*X*, where *M* = alkali, alkaline earth and rare earth metals, *T* = P, As, V, Si, S, B and *X* = F, Cl, OH, O, in hexagonal or pseudo-hexagonal symmetries. After the discovery of high oxide-ion conductivity in rare-earth orthosilicate oxyapatites (*T* = Si, *X* = O) by Nakayama *et al.* (1995), considerable attention has been drawn to related compounds for potential industrial applications.

In the title compound, the *M*1 site (site symmetry 3..) is occupied by Pr and Sr with almost even proportions, and surrounded by three O1, three O2 and three O3 atoms, forming a tri-capped trigonal prism. The *M* site (*m*.. symmetry) is almost exclusively occupied by Pr, and surrounded by one O1, one O2, four O3 and one O4 forming a distorted pentagonal bipyramid. The isolated SiO_4_ tetrahedra are composed of one O1, one O2 and two O3 atoms. They show a slight angular distortion and exhibit *m*.. symmetry. The O atom at the *X* site with 6.. symmetry is located at the centre of the *M*2 triangle and its anisotropic displacement ellipsoid is more than two times prolate along the *c* axis, a feature also observed for other members of the rare-earth orthosilicate oxyapatite family. The present compound is isostructural with CaLa_4_(SiO_4_)_3_O (Schroeder & Mathew, 1978).

### Experimental

All chemicals used were of analytical grade (3 N) from Kojundo Chemical Laboratory Co. Ltd., Japan. Powders of Pr_2_O_3_ (1.643 g), SiO_2_ (0.359 g) and SrCl_2_ (3.006 g) were mixed together and put into a platinum crucible (30 ml). The crucible capped with a platinum lid was then placed on alumina powder in an alumina crucible. The double crucible was heated in air to 1373 K at the rate of 100 K/h, held for 6 h at 1373 K, cooled at the rate of 20 K/h to 923 K, and then furnace-cooled by turning off the power. The flux components were washed away by distilled water in an oven at 373 K. Crystals were hexagonal prismatic with the longest dimension of 150 µm. Energy dispersive spectroscopy indicated that the crystals contained no elements other than Sr, Pr, Si and O. The Sr:Pr ratio was also estimated from eight samples to be 1.04:3.96 with an estimated standard uncertainty of ±0.06. Therefore, the stoichiometric composition of SrPr_4_Si_3_O_13_ was assumed for the refinement of X-ray data.

### Refinement

Possibility of lower symmetries like *P*6_3_ or *P*3 for the crystal was discarded in the course of refinements because no significant improvement was obtained. Defects at the O sites were not observed from the preliminary population analysis. Presence of interstitial O atoms were not detected in the difference Fourier maps. Populations of Sr and Pr at the *M*1 and *M*2 sites were refined with a restraint to satisfy the charge neutrality for the stoichiometric composition SrPr_4_Si_3_O_13_. The positional and atomic displacement parameters of Sr and Pr at both sites were constrained to unity, taking into account the respective site symmetries. The highest remaining peak is 0.45 Å from *M*2 and the deepest hole is 0.04 Å from *M*1.

### Crystal data

**Table:**

SrPr_4_(SiO_4_)_3_O	*D*_x_ = 5.5 Mg m^−^^3^
*M**_r_* = 943.53	Mo *K*α radiation, λ = 0.71073 Å
Hexagonal, *P*6_3_/*m*	Cell parameters from 9966 reflections
Hall symbol: -P 6c	θ = 4.3–61.7°
*a* = 9.5999 (1) Å	µ = 21.82 mm^−^^1^
*c* = 7.1388 (1) Å	*T* = 296 K
*V* = 569.76 (1) Å^3^	Prism, colourless
*Z* = 2	0.14 × 0.05 × 0.03 mm
*F*(000) = 840	

### Data collection

**Table:**

Bruker APEXII CCD diffractometer	3151 independent reflections
Radiation source: fine-focus sealed tube	2815 reflections with *I* > 2σ(*I*)
graphite	*R*_int_ = 0.030
φ and ω scans	θ_max_ = 62.0°, θ_min_ = 3.8°
Absorption correction: multi-scan (*SADABS*; Sheldrick, 1996)	*h* = −22→23
*T*_min_ = 0.240, *T*_max_ = 0.546	*k* = −23→23
31664 measured reflections	*l* = −17→17

### Refinement

**Table:**

Refinement on *F*^2^	2 constraints
Least-squares matrix: full	*w* = 1/[σ^2^(*F*_o_^2^) + (0.0249*P*)^2^ + 0.3254*P*] where *P* = (*F*_o_^2^ + 2*F*_c_^2^)/3
*R*[*F*^2^ > 2σ(*F*^2^)] = 0.018	(Δ/σ)_max_ = 0.001
*wR*(*F*^2^) = 0.047	Δρ_max_ = 2.45 e Å^−^^3^
*S* = 1.07	Δρ_min_ = −1.75 e Å^−^^3^
3151 reflections	Extinction correction: *SHELXL97* (Sheldrick, 2008), Fc^*^=kFc[1+0.001xFc^2^λ^3^/sin(2θ)]^-1/4^
42 parameters	Extinction coefficient: 0.0052 (3)
1 restraint	

### Special details

**Table:**

Geometry. All s.u.'s (except the s.u. in the dihedral angle between two l.s. planes) are estimated using the full covariance matrix. The cell s.u.'s are taken into account individually in the estimation of s.u.'s in distances, angles and torsion angles; correlations between s.u.'s in cell parameters are only used when they are defined by crystal symmetry. An approximate (isotropic) treatment of cell s.u.'s is used for estimating s.u.'s involving l.s. planes.
Refinement. Refinement of *F*^2^ against ALL reflections. The weighted *R*-factor *wR* and goodness of fit *S* are based on *F*^2^, conventional *R*-factors *R* are based on *F*, with *F* set to zero for negative *F*^2^. The threshold expression of *F*^2^ > 2σ(*F*^2^) is used only for calculating *R*-factors(gt) *etc*. and is not relevant to the choice of reflections for refinement. *R*-factors based on *F*^2^ are statistically about twice as large as those based on *F*, and *R*- factors based on ALL data will be even larger.

### Fractional atomic coordinates and isotropic or equivalent isotropic displacement parameters (Å^2^)

**Table:**

	*x*	*y*	*z*	*U*_iso_*/*U*_eq_	Occ. (<1)
Pr1	0.3333	0.6667	−0.000387 (15)	0.00860 (2)	0.5358 (8)
Sr1	0.3333	0.6667	−0.000387 (15)	0.00860 (2)	0.4641 (8)
Pr2	0.010753 (8)	0.242790 (8)	0.2500	0.00742 (2)	0.9761 (5)
Sr2	0.010753 (8)	0.242790 (8)	0.2500	0.00742 (2)	0.0239 (5)
Si1	0.39915 (5)	0.37061 (5)	0.2500	0.00683 (5)	
O1	0.31811 (17)	0.48323 (16)	0.2500	0.01330 (16)	
O2	0.59455 (14)	0.47298 (15)	0.2500	0.01224 (14)	
O3	0.34191 (14)	0.25210 (11)	0.06770 (12)	0.01501 (14)	
O4	0.0000	0.0000	0.2500	0.0152 (3)	

### Atomic displacement parameters (Å^2^)

**Table:**

	*U*^11^	*U*^22^	*U*^33^	*U*^12^	*U*^13^	*U*^23^
Pr1	0.00951 (3)	0.00951 (3)	0.00678 (3)	0.00476 (1)	0.000	0.000
Sr1	0.00951 (3)	0.00951 (3)	0.00678 (3)	0.00476 (1)	0.000	0.000
Pr2	0.00721 (2)	0.00712 (2)	0.00745 (2)	0.00323 (2)	0.000	0.000
Sr2	0.00721 (2)	0.00712 (2)	0.00745 (2)	0.00323 (2)	0.000	0.000
Si1	0.00702 (11)	0.00647 (11)	0.00747 (11)	0.00371 (10)	0.000	0.000
O1	0.0168 (4)	0.0127 (4)	0.0153 (4)	0.0110 (4)	0.000	0.000
O2	0.0079 (3)	0.0099 (3)	0.0171 (4)	0.0031 (3)	0.000	0.000
O3	0.0243 (4)	0.0119 (3)	0.0100 (2)	0.0098 (3)	−0.0065 (2)	−0.00319 (19)
O4	0.0107 (4)	0.0107 (4)	0.0242 (10)	0.00537 (19)	0.000	0.000

### Geometric parameters (Å, °)

**Table:**

Pr1—O1^i^	2.4617 (8)	Pr2—O3^vi^	2.4296 (8)
Pr1—O1^ii^	2.4617 (8)	Pr2—O3^iii^	2.4296 (8)
Pr1—O1	2.4617 (8)	Pr2—O2^i^	2.4568 (12)
Pr1—O2^iii^	2.5258 (9)	Pr2—O3^vii^	2.5519 (10)
Pr1—O2^iv^	2.5258 (9)	Pr2—O3^viii^	2.5519 (10)
Pr1—O2^v^	2.5258 (9)	Pr2—O1	2.6876 (15)
Pr1—O3^iv^	2.8509 (12)	Si1—O1	1.6172 (12)
Pr1—O3^iii^	2.8509 (12)	Si1—O2	1.6251 (13)
Pr1—O3^v^	2.8509 (12)	Si1—O3	1.6324 (9)
Pr2—O4	2.2809 (1)	Si1—O3^ix^	1.6324 (9)
			
O1—Si1—O2	113.04 (7)	O1—Si1—O3^ix^	111.05 (5)
O1—Si1—O3	111.05 (5)	O2—Si1—O3^ix^	107.82 (5)
O2—Si1—O3	107.82 (5)	O3—Si1—O3^ix^	105.73 (7)

Symmetry codes: (i) −*x*+*y*, −*x*+1, *z*; (ii) −*y*+1, *x*−*y*+1, *z*; (iii) *x*−*y*, *x*, −*z*; (iv) *y*, −*x*+*y*+1, −*z*; (v) −*x*+1, −*y*+1, −*z*; (vi) *x*−*y*, *x*, *z*+1/2; (vii) −*y*, *x*−*y*, *z*; (viii) −*y*, *x*−*y*, −*z*+1/2; (ix) *x*, *y*, −*z*+1/2.
